# Prevalence and epidemiological characteristics of patients with diabetic retinopathy in Slovakia: 12-month results from the DIARET SK study

**DOI:** 10.1371/journal.pone.0223788

**Published:** 2019-12-12

**Authors:** Marta Ondrejkova, Peter Jackuliak, Emil Martinka, Marian Mokan, James Foley, Jana Fabkova, Karol Gecik, Iveta Tvrda, Miroslav Helbich, Monika Gajdosova, on behalf of the DIARET SK study group

**Affiliations:** 1 Specialized Hospital in Ophthalmology, Zvolen, Slovakia; 2 5^th^ Clinic of Internal Medicine, Faculty of Medicine of the Comenius University Bratislava and University Hospital Bratislava, Workplace Hospital, Ruzinov, Slovakia; 3 National Institute of Endocrinology and Diabetology, Lubochna, Slovakia; 4 1^st^ Clinic of Internal Medicine, Jessenius Faculty of Medicine in Martin of the Comenius University in Bratislava, Slovakia; 5 Novartis Pharmaceuticals Corporation, East Hanover, New Jersey, United States of America; 6 Novartis Slovakia s.r.o., Bratislava, Slovakia; 7 Caldera s.r.o. Banska Stiavnica, Banska Stiavnica, Slovakia; Medical School, University of Zagreb, CROATIA

## Abstract

**Purpose:**

To evaluate the prevalence and epidemiological characteristics of diabetic retinopathy (DR) in Slovakian patients with Type 1 and 2 diabetes mellitus (DM) in the DIARET SK study.

**Patients and methods:**

An epidemiological multi-center survey that included 4,078 adult patients (aged ≥18 years) from 51 diabetologists and 47 ophthalmologists. Data were collected from February to December 2015.

**Results:**

The final data set consisted of 4,014 patients; 3,700 were enrolled (Type 2 DM = 3,405, Type 1 DM = 295) using a quasi-random approach; 16 (Type 2 DM = 15, Type 1 DM = 1) patients in the pre-specified group had DM duration of <5 years with a history of DR while 298 patients (Type 2 DM = 204, Type 1 DM = 94) had DM duration of ≥ 20 years. The mean (standard deviation [SD]) age of patients at diagnosis for Types 2 and 1 DM was 53.4 (9.5) and 27.6 (12.9) years, respectively. The mean (SD) glycated hemoglobin (HbA1c) was 7.5 (1.4) and 8.5 (1.6) in Types 2 and 1 DM patients, respectively, whereas a slightly higher proportion of patients had >11.0 HbA1c in Type 1 DM (5.8%) than Type 2 (2.0%). The mean (SD) duration of Type 2 DM was shorter compared with Type 1 (7.5 [5.2] vs 10.3 [6.9] years). In Type 2 DM patients, there were 516 (15.5%) cases of DR, 19 (0.56%) of proliferative DR (PDR), and 106 (3.11%) of diabetic macular edema (DME). In Type 1 DM patients, there were 86 (29.15%) cases of DR, 10 (3.39%) PDR, and 12 (4.07%) DME.

**Conclusions:**

In Slovakian patients with DM, the duration of disease and higher HbA1c were the most prevalent factors that contributed to the development of DR and DME.

## Introduction

Diabetes is one of the most prevalent health disorders of the 21^st^ century. The majority of people suffering from diabetes are from low income or developing countries [1–2]. According to the International Diabetes Federation 2017 report, there are currently nearly 425 million people aged 20–79 years who have the condition [1–2]. By 2045, nearly 693 million people will be suffering with diabetes worldwide [3]. Diabetic retinopathy (DR) accounts for 4.8% of blindness throughout [4] the world and has a global prevalence of 34.6% [5] and is mainly found in patients aged between 20–74 years [6, 7]. It is predicted that between 2010 and 2030, developing countries will see a 69% increase in the number of adult diabetic patients with a corresponding 20% increase in the developed countries [2]. DR is classified into two types: non-proliferative diabetic retinopathy (NPDR) and proliferative DR (PDR). PDR is an advanced stage of DR and occurs due to abnormal angiogenesis on the surface of the retina [6, 8]. The prevalence of DR and PDR is higher amongst people with Type 1 diabetes mellitus (DM) compared with Type 2 [5]. In patients with DM, DR is the leading cause of blindness, but the data available on the prevalence of DR are inconsistent.

To date, there are no data on the prevalence of DR and its stages or on diabetic macular edema (DME) in Slovakia. Here, we present the first data on the prevalence and epidemiological characteristics in Slovakian diabetic patients from the “Prevalence of **DIA**betic **RET**inopathy and impact of genetic factors in the development of Diabetic Retinopathy of patients with Type 1 and 2 DM in **S**lova**K**ia” (DIARET SK) study.

## Materials and methods

### Study design

DIARET SK (NCT02232503) was an epidemiological, multi-center survey in adult patients (aged ≥18 years) with Types 1 and 2 DM who fulfilled the eligibility criteria and signed the informed consent form for epidemiological research. The first visit was at the patient’s diabetologist during regular visit where they were examined for required parameters that were recorded electronically with retrospective anamnestic data. Each enrolled patient was referred to the ophthalmologist (second visit) who performed examination of both eyes for best-corrected visual acuity (BCVA), by slit lamp biomicroscopy and optical coherence tomography (OCT). Fundus photographs (FP) were taken for the evaluation of the presence and signs of DR and DME. The data were recorded electronically using secure software.

**Fig 1:**
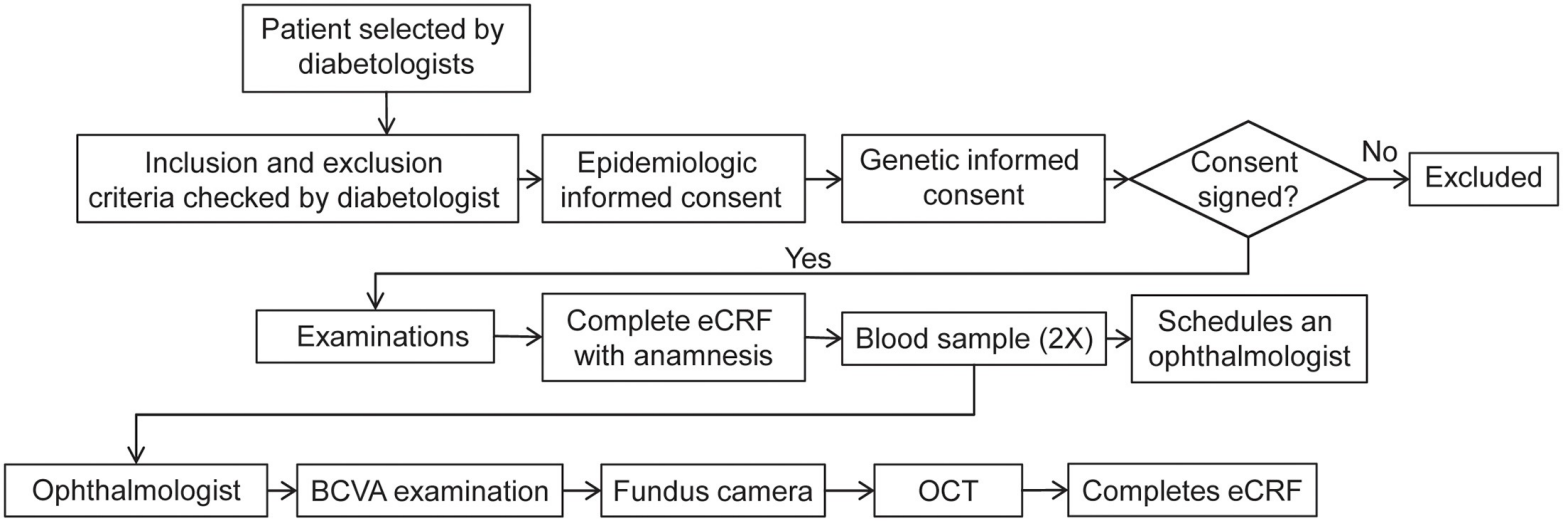
Patient flow chart. All diabetic patients were included, irrespective of disease type. BCVA, best-corrected visual acuity; DM, diabetes mellitus; eCRF, electronic case report form; OCT, optical coherence tomography.

Patients were assigned a unique identity code to maintain anonymity and the patient identity was only known to the attending physician (Fig 1). The data were collected from 51 diabetologists and 47 ophthalmologists from February to December 2015. The study was conducted in accordance with the Declaration of Helsinki and the Guidelines for Good Pharmacoepidemiology Practice. The study was reviewed and approved by institutional review board/ethics committee—Etická komisia Bratislavského samosprávneho kraja, before the study began. Signed informed consent for epidemiological and genetic research was obtained from each patient. The study is registered with clinicaltrials.gov as NCT02232503.

### Patients

To ensure non-biased selection, patients were selected on each screening day in a pre-specified sequence. All selected patients with DM (Type 1 and 2) were included regardless of DM duration and of the eye complications in a patient’s anamnesis or during diabetologist examination.

Patients were excluded if they 1) were <18 years of age, 2) had gestational or secondary-induced diabetes, 3) had diabetic ketoacidosis, or hyperosmolar coma, and 4) had history of alcohol abuse or acute alcohol intoxication.

### Objectives

The primary objective of the study was to evaluate the prevalence of DR in patients with Types 2 and 1 DM according to DM duration.

The secondary objectives were to evaluate the prevalence and individual stages of DR and DME in patients with Types 2 and 1 DM based on complex ophthalmologic examinations.

Another objective was to obtain the epidemiological characteristics of patients with DM and DR in terms of socio/demographic structure, treatment, and control of DM, and the presence of other microvascular and ophthalmologic complications.

Physical examinations of patients were performed by the diabetologists whereas BCVA, OCT, FP, and other ophthalmological examinations were performed by ophthalmologists.

### Safety

Due to the epidemiological nature of the study without follow-up and study treatment, no adverse drug reactions or adverse events were required to be collected according to the study protocol and Slovak legislation rules. Physicians reported adverse drug reactions in line with Slovakian regulations to the relevant National Health Authority. No safety events were reported in this study.

### Statistical analysis

The total expected number of enrolled patients was 5,000. The initial study protocol was approved for 4,500 patients to be enrolled by a pre-defined quasi-random selection process to overcome the weakness of the non-biased random selection. Overall, 3,700 patients were enrolled by this random selection. To ensure a sufficient sample size of less frequent groups of patients for statistical analysis, all patients from pre-specified groups were enrolled in the study even if they were out of pre-specified sequence. A pool of 500 patients was reserved for special subgroups: patients with DM duration of ≥20 years and patients with DM duration of <5 years with DR in their history. Although data from fewer patients were collected, the sample size was sufficient to fulfill the primary and secondary objectives of this epidemiological study. Similar published data [9–11] worked with smaller populations. Because of the high number of patients, precise results in term of statistical error were expected, including total prevalence and identification of risk factors contributing to the development of DR.

The primary endpoint results were accompanied by a Wilson score with 95% confidence interval (CI). The calculation of prevalence for each stage of DR and DME were analyzed using the same methods as for the total DR prevalence. The analysis of the impact of risk factors on prevalence of DR and DME was carried out using multivariate logistics regression. The rate of missing values was generally low. There was no imputation of missing data. The rate of missing data was presented for each parameter where appropriate. In analyses of correlations or logistics regression all data with available results were used.

Age, DM duration since diagnosis, diabetes control based on the average glycated hemoglobin (HbA1c) of all measurements in the past 12 months and body mass index (BMI) were assessed as continuous covariates whereas sex, nationality, presence of nephropathy, hypertension and dyslipidemia were considered categorical variables.

The patient characteristics were described using the standard methods of descriptive statistics: total number of patients (N), percentage (%), mean, median, minimum, maximum, standard deviation (SD) and, where necessary, accompanied by the histogram or contingence table.

## Results

The data of 4,078 patients were collected from 51 diabetologists and 47 ophthalmologists. Data from 64 patients were excluded from the analysis (3 had incomplete diabetological examinations, 50 had no ophthalmological examinations, and 11 were missing demographic data). The final data set consisted of data from 4,014 patients.

Using a quasi-random approach, 3,700 patients (Type 2 DM = 3405, Type 1 DM = 295), 16 (Type 2 DM = 15, Type 1 DM = 1) were enrolled in the pre-specified group with a duration of DM of <5 years with a DR history, and 298 (Type 2 DM = 204, Type 1 DM = 94) patients with a duration of DM of ≥20 years. Results are presented separately according to DM type.

**Fig 2:**
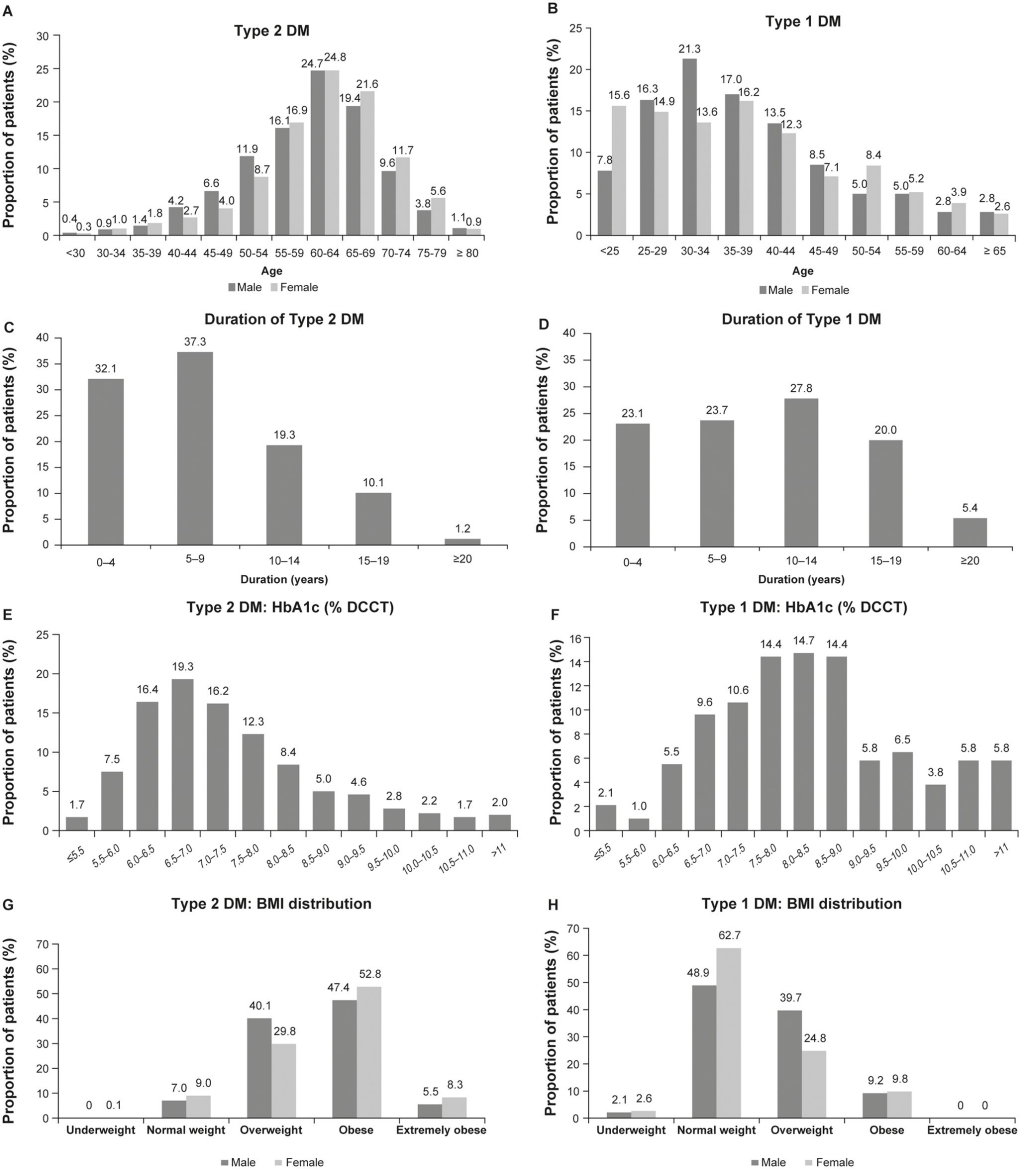
A. Age distribution of Type 2 DM patients B. Age distribution of Type 1 DM patients C. Duration of Type 2 DM D. Duration of Type 1 DM E. Mean HbA1c levels in Types 2 DM patients F. Mean HbA1c levels in Types 1 DM patients G. BMI distribution of Types 2 DM patients H. BMI distribution of Types 1 DM patients BMI, body mass index; DCCT, Diabetes control and complications trial; DM, diabetes mellitus; HbA1c, glycated hemoglobin.

The proportion of female patients was similar between both Types 2 and 1 DM groups (1806 [53.0%) vs 154 [52.2%], respectively). The mean age (SD) at the time of DM diagnosis was 53.4 (9.5) years and 27.6 (12.9) years for Types 2 and 1 DM patients, respectively. The mean age (SD) of the patients was 60.9 (9.5) years in Type 2 DM patients and 37.9 (12.1) years in Type 1 DM patients. Most of the patients in the Type 2 DM group were between 50–70 years, whereas in the Type 1 DM group, they were under 45 years (Fig 2A and 2B). In the overall study population, the best corrected visual acuity (BCVA) of the study eye was 79.6 ETDRS letters (median 83.0, minimum 0, maximum 100). No clinically significant difference in BCVA between Type 2 DM patients (mean 78.9, median 83.0) and Type 1 DM patients (mean 81.3, median 84.0) was observed.

The mean (SD) duration of Types 2 and 1 DM was 7.5 (5.2) and 10.3 (6.9) years. The disease duration of Type 2 DM in the majority (69.4%) of patients was <10 years and ≥20 years in only 40 (1.2%) patients. Of all the Type 1 DM patients, 46.8% had disease duration of <10 years and only 5.4% had disease duration ≥20 years. The duration of Type 2 and 1 DM is shown in Fig 2C and 2D.

The prevalence of DR was found to be higher in patients with Type 2 and 1 DM of duration ≥20 years compared with DM of duration <20 years (**primary endpoint**; Table 1).

**Table 1:** Prevalence of any DR according to DM duration and type.

Duration (years)	Type 2 DM			Type 1 DM		
	N	n	Prevalence per 100 (95% CI)*	N	n	Prevalence per 100 (95% CI)*
**0–4**	1,092	65	5.95 (4.7; 7.52)	68	7	10.29 (5.08; 19.76)
**5–9**	1,270	157	12.36 (10.66; 14.29)	70	10	14.29 (7.95; 24.34)
**10–14**	658	148	22.49 (19.47; 25.84)	82	30	36.59 (26.98; 47.39)
**15–19**	345	122	35.36 (30.5; 40.54)	59	27	45.76 (33.7; 58.34)
**20–24**	144 (30+114)	72 (19+53)	50 (41.94; 58.06)	42 (8+34)	26 (6+20)	61.9 (46.81; 75)
**25–29**	56 (4+52)	36 (2+34)	64.29 (51.19; 75.54)	27 (4+23)	16 (3+13)	59.26 (40.73; 75.49)
**≥ 30**	44 (6+38)	25 (3+22)	56.82 (42.22; 70.32)	41 (4+37)	27 (3+24)	65.85 (50.55; 78.44)

*95% CI = 95% Wilson score interval

The number of cases from random selected patients and patients in predefined subgroup with duration of DM ≥ 20 years are shown separately in parentheses. CI, confidence interval; DM, diabetes mellitus; DR, diabetic retinopathy; N, total number of patients; n, number of patients

The prevalence of PDR was also found to be higher in patients with DM duration ≥20 years compared with DM duration <20 years, irrespective of DM type. Compared with Type 2 DM, the prevalence of PDR was higher in Type 1 DM patients with a disease duration of ≥20 years (Table 2).

**Table 2:** Prevalence of PDR according to DM duration and type.

Duration (years)	Type 2 DM			Type 1 DM		
	N	n	Prevalence per 100 (95% CI)*	N	n	Prevalence per 100 (95% CI)*
**0–4**	1,092	1	0.09 (0.02; 0.52)	68	1	1.47 (0.26; 7.87)
**5–9**	1,270	4	0.31 (0.12; 0.81)	70	2	2.86 (0.79; 9.83)
**10–14**	658	5	0.76 (0.32; 1.77)	82	3	3.66 (1.25; 10.21)
**15–19**	345	7	2.03 (0.99; 4.13)	59	1	1.69 (0.3; 9)
**≥ 20**	244 (40+204)	10 (2+8)	4.1 (2.24; 7.38)	110 (16+94)	10 (3+7)	9.09 (5.01; 15.93)

*95% CI = 95% Wilson score interval

The number of cases from random selected patients and patients in predefined subgroup with duration of DM ≥ 20 years are shown separately in parentheses. CI, confidence interval; DM, diabetes mellitus; N, total number of patients; n, number of patients; PDR, proliferative diabetic retinopathy

DME was found to be most prevalent amongst Type 2 DM patients with a disease duration of >20 years. These data suggest a steady rise of DME prevalence according to DM duration in both types of DM (Table 3).

**Table 3:** Prevalence of DME according to DM duration and type.

Duration (years)	Type 2 DM			Type 1 DM		
	N	n	Prevalence per 100 (95% CI)*	N	n	Prevalence per 100 (95% CI)*
**0–4**	1,092	10	0.92 (0.5; 1.68)	68	1	1.47 (0.26; 7.87)
**5–9**	1,270	10	1.89 (1.27; 2.8)	70	3	4.29 (1.47; 11.86)
**10–14**	658	10	5.02 (3.59; 6.96)	82	3	3.66 (1.25; 10.21)
**15–19**	345	10	9.57 (6.89; 13.13)	59	4	6.78 (2.67; 16.18)
**≥ 20**	244 (40+204)	43 (6+37)	17.62 (13.35; 22.89)	110 (16+94)	10 (1+9)	9.09 (5.01; 15.93)

*95% CI = 95% Wilson score interval

The number of cases from random selected patients and patients in predefined subgroup with duration of DM ≥ 20 years are shown separately in parentheses. CI, confidence interval; DM, diabetes mellitus; DME, diabetic macular edema; N, total number of patients; n, number of patients

The overall prevalence of any DR and vision-threatening DR (VTDR) in Type 2 DM patients was 15.15% and 3.35%, respectively. Male patients had a slightly higher prevalence of DR irrespective of type (16.70%) compared with female patients (13.79%) (Table 4).

**Table 4:** Overall prevalence of diabetic retinopathy in patients with Type 2 DM.

Type 2 DM	Total (N)	Cases (n)	Prevalence per 100 (95% CI)*	
**All**				
Any DR	3,405	516	15.15	(13.95; 16.36)
PDR	3,405	19	0.56	(0.31; 0.81)
DME	3,405	106	3.11	(2.53; 3.7)
VTDR	3,405	114	3.35	(2.74; 3.95)
**Men**				
Any DR	1,599	267	16.70	(14.87; 18.53)
PDR	1,599	11	0.69	(0.28; 1.09)
DME	1,599	57	3.56	(2.66; 4.47)
VTDR	1,599	60	3.75	(2.82; 4.68)
**Women**				
Any DR	1,806	249	13.79	(12.2; 15.38)
PDR	1,806	8	0.44	(0.14; 0.75)
DME	1,806	49	2.71	(1.96; 3.46)
VTDR	1,806	54	2.99	(2.2; 3.78)

*95% CI = 95% Wilson score interval

DM, diabetes mellitus; DME, diabetic macular edema; DR, diabetic retinopathy; N, total number of patients; n, number of patients; PDR, proliferative diabetic retinopathy; VTDR, vision threatening diabetic retinopathy

The overall prevalence of any DR in Type 1 DM patients was 29.15% (Table 5).

**Table 5:** Overall prevalence of diabetic retinopathy in patients with Type 1 DM.

Type 1 DM	Total (N)	Cases (n)	Prevalence per 100 (95% CI)*	
**All**				
Any DR	295	86	29.15	(23.97; 34.34)
PDR	295	10	3.39	(1.32; 5.45)
DME	295	12	4.07	(1.81; 6.32)
VTDR	295	17	5.76	(3.1; 8.42)
**Men**				
Any DR	141	42	29.79	(22.24; 37.34)
PDR	141	5	3.55	(0.49; 6.6)
DME	141	5	3.55	(0.49; 6.6)
VTDR	141	8	5.67	(1.86; 9.49)
**Women**				
Any DR	154	44	28.57	(21.44; 35.71)
PDR	154	5	3.25	(0.45; 6.05)
DME	154	7	4.55	(1.26; 7.84)
VTDR	154	9	5.84	(2.14; 9.55)

*95% CI = 95% Wilson score interval

CI, confidence interval; DM, diabetes mellitus; DME, diabetic macular edema; DR, diabetic retinopathy; N, total number of patients; n, number of patients; PDR, proliferative diabetic retinopathy; VTDR, vision threatening diabetic retinopathy

### Epidemiological characteristics of patients with diabetes and diabetic retinopathy

The mean (SD) HbA1c was 7.5 (1.4) in Type 2 DM patients and 8.5 (1.6) in Type 1 DM patients. HbA1c levels in the majority of Type 2 DM patients (72.6%) ranged between 6.0–8.5%, whereas HbA1c levels in the majority of Type 1 DM patients (63.7%) ranged between 6.5–9.0%. Overall, 55.2% of patients with Type 2 DM and 81.8% of patients with Type 1 DM had HbA1c levels above the normal range (>7%)[12]. Two percent of the patients with Type 2 DM and 5.8% of the patients with Type 1 DM had HbA1C >11 (Fig 2E and 2F).

The mean (SD) BMI was 31.5 (5.3) kg/m^2^ in Type 2 DM patients and 24.7 (4.0) kg/m^2^ in Type 1 patients. The BMI was higher for male patients in both DM types compared with female patients (Fig 2G and 2H). The majority of Type 2 DM patients were overweight (males: 40.1%, females: 29.8%) or obese (males: 47.4%, females: 52.8%). In the Type 1 DM group, nearly 50% of the male patients and 62.7% of the female patients were of normal weight. A total of 467 (13.7%) patients in Type 2 and 54 (18.3%) in Type 1 DM groups were smokers. The proportion of ex-smokers was 525 (15.4%) and 19 (6.4%) in the Type 2 and 1 DM groups, respectively.

Sex distribution in Type 2 and 1 DM with DR was similar. The mean (SD) age of Type 2 and 1 DM patients was 61.7 (8.6) and 39.2 (13.5) years, respectively. Duration of DM in Type 2 and 1 patients was 10.9 (5.7) and 14.4 (7.3) years. Other epidemiological characteristics of patients are shown in Table 6.

**Table 6:** Epidemiological characteristics of patients with DR.

	Type 2 DM (n = 516)	Type 1 DM (n = 86)
Sex, Female, n (%)	249 (48.3)	44 (51.2)
Age (mean ± SD), years	61.7 ± 8.6	39.2 ± 13.5
Duration of DM (median ± SD), years	7 ± 5.2	10 ± 6.9
**Treatment, n (%)**		
Only diet	18 (3.5)	0 (0.0)
OAD	423 (82.0)	3 (3.5)
Insulin	257 (49.8)	86 (100.0)
**Glycemic control, n (%)**		
Satisfactory	250 (48.4)	21 (24.4)
Unsatisfactory	266 (51.6)	65 (75.6)
**HbA1c (% DCCT)**		
≤ 5.5	5 (1.0)	0 (0.0)
5.5–6.0	18 (3.6)	0 (0.0)
6.0–6.5	35 (6.9)	3 (3.5)
6.5–7.0	64 (12.7)	5 (5.8)
7.0–7.5	67 (13.3)	3 (9.3)
7.5–8.0	77 (15.2)	8 (9.3)
8.0–8.5	46 (9.1)	12 (14.0)
8.5–9.0	44 (8.7)	16 (18.6)
9.0–9.5	47 (9.3)	9 (10.5)
9.5–10.0	24 (4.8)	11 (12.8)
10.0–10.5	23 (4.6)	4 (4.7)
10.5–11.0	19 (3.8)	6 (7.0)
> 11.0	36 (7.1)	9 (10.5)
HbA1c (mean ± SD)	8.3 ± 1.6	9.2 ± 1.7
**Other DM complications, n (%)**		
Any DM complication (without DR)	319 (61.8)	64 (74.4)
Diabetic neuropathy	230 (44.6)	48 (55.8)
Diabetic nephropathy	117 (22.7)	33 (38.4)
Ischemic heart disease	111 (21.5)	4 (4.7)
Peripheral vascular disease	58 (11.2)	5 (5.8)
Stroke	29 (5.6)	1 (1.2)
Chronic heart failure	9 (1.7)	1 (1.2)

DCCT, diabetes control and complications trial; DM, diabetes mellitus; DR, diabetic retinopathy; HbA1c, glycated hemoglobin; N, number of patients; n, number of patients; OAD, oral anti–diabetic drugs; SD, standard deviation

## Discussion

DIARET SK is the first large study in Slovakia assessing the prevalence of DR and DME, their epidemiological characteristics, and the impact of these on the development of DR and DME. DR is a key indicator of microvascular complications associated with DM. The prevalence of DM increases with increasing age [13], resulting in DR-related complications. In the current study, the mean age of the patients with Type 2 DM was higher than in Type 1 DM, and our results are similar to those reported in a recent Danish study in which patients data were collected from the Odense University Hospital and stored at Funen Diabetes Database [14].

In this study, the prevalence of any DR was 15.5%, and that of VTDR was 3.35% in patients with Type 2 DM. In Type 1 DM patients, the prevalence of any DR was 29.15%, and that of VTDR was 5.76%. These numbers are low compared with the prevalence of DR observed in both the Swedish and Danish populations. In the Swedish study (population based), the prevalence of any DR was 27.9% and 41.8% in Type 2 and Type 1 DM patients whereas, in the Danish study, it was 21.2% and 54.3% in Type 2 and Type 1 DM patients, respectively [14, 15]. Results from a systemic screening carried out in Liverpool (primary care-based) showed the proportion of patients with DR and VTDR to be 25.3% and 6.0% in Type 2 DM patients and 45.7% and 16.0% in Type 1 DM patients, respectively [16]. From a national screening carried out in Wales (community-based mobile screening service), the prevalence of any DR and VTDR in patients with Type 2 DM was 30.3% and 2.9% and in those with Type 1 DM was 56.0% and 11.2%, respectively [17]. Results from a recently carried out population based study in Spain by Romero-Aroca, et al. showed a similar incidence of DR and VTDR in Type 2 and Type 1 DM patients. Compared with Type 2 DM patients, a higher incidence of DR and VTDR was observed over a span of 9 years in Type 1 DM patients who had a longer duration of DM [18].

The above results are interesting when compared with the DIARET SK study with respect to the median duration of DM. Median durations of diabetes in patients with Types 2 and 1 DM were 3.2 and 12.8 years, respectively, in the Liverpool study; 5.3 and 16.7 years, respectively, in the Wales study; and 8 and 19 years, respectively, in the Danish study. In our study, the median duration of DM was 7 years in Type 2 DM patients and 10 years in Type 1 DM patients. The majority of Type 2 DM patients (69.4%) had a disease duration of <10 years and only 30.6% and 53.2% of patients had a disease duration of >10 years in Types 2 and 1 DM, respectively. This could be one of the reasons for the higher prevalence of DR amongst Type 1 DM patients in this study, similar to what has been observed in previously published studies [16–18]. These results indicate that the prevalence of DR is directly proportional to the duration of DM.

The data from DIARET SK suggest that male patients have a slightly higher prevalence of DR, irrespective of the type of disease, compared with female patients (16.70 vs 13.79). Similar findings were also reported in the Liverpool (Odds ratio [OR] male patients: 2.15, 95% CI 1.39–3.31; P = 0.001), Wales (Type 2 DM: 59.1% vs 40.9%; Type 1 DM: 54.7% vs 45.3%), and Swedish (30.9% vs 27.4%) studies [15–17].

In our study, DME was highly prevalent in patients with a DM duration of >15 years, irrespective of the DM types. Prevalence of DME varies in previously published population-based studies. In a Danish study published in 2006, DME was prevalent in 12.8% of Type 2 and 7.9% of Type 1 DM patients [19], prevalence was 7.6% in both Type 2 and 1 DM patients in a literature based survey carried out in Australian indigenous population [20], 15.7% in Type 2 and Type 1 patients in a longitudinal population-based database study from Canada [21], and 3.9% in both Type 2 and Type 1 DM patients in a population-based multipurpose study from Norway [22]. Results from our study show a DME prevalence between 0.92%– 17.62% in Type 2 DM patients and between 1.47%– 9.09% in Type 1 DM patients.

HbA1c represents chronic blood glucose concentration, acts as a marker to predict future diabetes-related complications, and is a critical parameter for the assessment of disease impact [23]. Several clinical trials have been carried out to study the effect of lowered blood glucose levels on microvascular complications in Types 2 and 1 DM patients. Results from these studies indicate that a chronic reduction in blood glucose levels subsequently reduced the risk of retinopathy [24–29]. Results from the Diabetes Control and Complications Trial (DCCT) in the US and Canada for Type 1 DM and the United Kingdom Prospective Diabetes Study (UKPDS) for Type 2 DM demonstrate highly significant reductions (DCCT, p<0.001; UKPDS, p = 0.009) in the incidence and progression of retinopathy in patients randomized to tight blood glucose control (HbA1c <7%). However, results from the ADVANCE and ACCORD studies demonstrated that aggressive glycemic control (<6.5%) did not significantly reduce the risk of retinopathy [30, 31]. In DIARET SK, irrespective of the type of DM, the majority of patients had mean HbA1c levels above the normal range (>7.0%). The mean HbA1c levels were higher in Type 1 DM patients compared with those of Type 2 patients which could be one of the reasons for a higher prevalence of DR in Type 1 DM patients in DIARET SK. These results from DIARET SK are similar to what has been reported in the Spanish population (mean [SD]: 7.38±1.29% in Type 2 DM and 8.38±1.16% in Type 1 DM patients) [18]. Surprisingly, in our study, although the Type 1 DM patients have higher mean HbA1c levels compared with Type 2 DM patients, the prevalence of DME was comparatively higher in Type 2 DM patients. Similar to the higher HbA1c levels, the mean BMI was also higher in male compared with female patients in the DIARET SK study. However, results from a recent meta-analysis indicate that a higher BMI is not significantly associated with an increased risk of DR [32].

DIARET SK is the first large, well-controlled epidemiological study that evaluated the prevalence of DR based on FP and OCT. The use of OCT is one of the strengths of the study because it is more precise for the diagnosis of DME than FP. Therefore, DME data included in this study are reliable and accurate. To the best of our knowledge, no other epidemiological study with such a high number of patients using OCT for diagnosis of DME has been published to date. The other main strength of this study is the large sample of patients, which allowed us to present the data in the finest of intervals according to disease duration. Despite the fact that the study was epidemiological with a real-life clinical practice setting, it was monitored on-site and electronically by an external clinical research organization. Reconciliation of diagnosis was done by independent retinal specialists in 10% of patients based on their FP. Although DIARET SK was a well-controlled epidemiological study which was designed to provide minimum bias, there was always a possibility to do better in terms of bias reduction by reducing the target area and increasing the coverage of included patients. However, this approach was not possible in Slovakian setting since the pressure on collecting the required large number of patients in short time was at its limit. Though a large number of patients were recruited into the DIARET SK study, subject enrollment took place at the outpatient offices which may not reflect the true epidemiological and population-based parameters of the disease. The amount of missing data was very low and did not significantly impact on the results.

Outpatient healthcare in Slovakia is based on the general practitioner offices network. General practitioners actively screen for diabetes in patients at risk. All patients with confirmed diabetes mellitus are referred to diabetes specialist who resumes further management of diabetes and searches for the presence of diabetic complications in collaboration with other specialists. Initial evaluation by ophthalmologist is performed early as the diabetes mellitus diagnosis is confirmed and subsequent follow up is planned and eye treatment initiated based on the initial eye finding and its development. Taking into account this active screening system employed in the Slovakian healthcare, we believe the data collected are very accurate.

The interpretation of the current study results should be done in the context of other previously published results. Large sample of patients included in this study allowed us to present data in the finest intervals according to disease duration and other factors. Results from the DIARET SK study reiterate the fact that focus should be on good control of HbA1c, blood pressure and lipids as they present the main manageable risk factors that contribute to the development of DR.

In conclusion, this epidemiological study from the Slovakian population confirms the results from previous studies in other populations showing that glycemic exposure (the duration of DM and HbA1c) is the predominant factor for the development of DR. Good control of HbA1c and earlier diagnosis of DM would help in better managing these patients with a reduced treatment burden. Data from DIARET SK would provide the basis for comparison against various other studies in order to gain a full insight on the management and better understanding of this vision threatening disease.

## Supporting information

S1 STROBE Statement Checklist of items that should be included in reports of observational studies. (DOCX)
